# A Draft Map of the Human Ovarian Proteome for Tissue Engineering and Clinical Applications

**DOI:** 10.1074/mcp.RA117.000469

**Published:** 2018-02-23

**Authors:** Emna Ouni, Didier Vertommen, Maria Costanza Chiti, Marie-Madeleine Dolmans, Christiani A. Amorim

**Affiliations:** From the ‡Pôle de Recherche en Gynécologie, Institut de Recherche Expérimentale et Clinique, Université Catholique de Louvain, Brussels, Belgium;; §de Duve Institute, Université Catholique de Louvain, Brussels, Belgium;; ¶Gynecology Department, Cliniques Universitaires Saint-Luc, Brussels, Belgium

**Keywords:** Extracellular matrix*, Ontology*, Immunohistochemistry, Label-free quantification, Mass Spectrometry, Fertility preservation, Matrisome, Ovarian follicle, Ovary

## Abstract

Fertility preservation research in women today is increasingly taking advantage of bioengineering techniques to develop new biomimetic materials and solutions to safeguard ovarian cell function and microenvironment *in vitro*, and *in vivo*,. However, available data on the human ovary are limited and fundamental differences between animal models and humans are hampering researchers in their quest for more extensive knowledge of human ovarian physiology and key reproductive proteins that need to be preserved. We therefore turned to multi-dimensional label-free mass spectrometry to analyze human ovarian cortex, as it is a high-throughput and conclusive technique providing information on the proteomic composition of complex tissues like the ovary. In-depth proteomic profiling through two-dimensional liquid chromatography-mass spectrometry, Western blotting, histological and immunohistochemical analyses, and data mining helped us to confidently identify 1508 proteins. Moreover, our method allowed us to chart the most complete representation so far of the ovarian matrisome, defined as the ensemble of extracellular matrix proteins and associated factors, including more than 80 proteins. In conclusion, this study will provide a better understanding of ovarian proteomics, with a detailed characterization of the ovarian follicle microenvironment, in order to enable bioengineers to create biomimetic scaffolds for transplantation and three-dimensional *in vitro*, culture. By publishing our proteomic data, we also hope to contribute to accelerating biomedical research into ovarian health and disease in general.

The World Health Organization has ranked infertility in women as the fifth highest serious global disability (1). Based on this critical assessment, several strategies have been developed to preserve and even restore fertility in women.

Although the human ovary is relatively well understood in terms of secretory patterns of ovarian hormones and the pathogenesis of ovarian diseases, little is known about the molecular composition and regulation of the microenvironment that directs the development and function of ovarian follicles, the functional units that mainly reside in the ovarian cortex and play an important role in oogenesis and gonadal hormone secretion. A few studies have focused on mRNA expression to provide a complete characterization of gene expression in the ovary. However, study of gene expression at the mRNA level yields no information about post-transcriptional modifications (2). Moreover, even abundant mRNA transcripts may be translated inefficiently or degrade rapidly, resulting in lower than expected levels of protein expression (3).

On the other hand, by focusing on final gene products, a proteomic approach has the advantage of investigating complex biological events and diseases, providing more conclusive information.

For several years now, ovarian proteomic studies have concentrated on characterizing follicular fluid composition in human preovulatory follicles (4–5), emphasizing the functional selectivity of the basement membrane toward plasma proteins (6). Efforts have also been made to identify ovary-related transcription in different species to explore the complex functions of the ovary in an integrated manner. This is necessary because of obvious difficulties in obtaining human ovaries, as well as the feasibility of conducting experimental research in humans because of ethical and logistical constraints. He *et al.*, (2015). described ovarian proteomics in rhesus monkeys through identification of 4325 proteins to provide a basis for future studies of human reproductive disorders using this animal as a model (7). To our knowledge, the only proteomic analysis of human ovaries performed to date was conducted by Wang *et al.*, (2005) (8). The study identified 138 proteins by two-dimensional (2D) electrophoresis and MALDI-TOF mass spectrometry, but without differentiation of ovarian extracellular matrix (ECM)^1^ proteins, despite the ECM's important role in defining the follicular environment and orchestrating cellular organization and function. Indeed, the only aspect of the ovarian ECM to have been investigated so far is the basement follicular membrane, and this was done through immunohistochemical tests, which only offer a limited characterization restricted by the type and number of antibodies used (9–10).

The goal of this study was therefore to provide a draft map of functional proteins identified in human ovarian cortex and complement available ovarian ECM data. By using 2D liquid chromatography-mass spectrometry (2D-LC/MS) and ensuring adequate sample preparation, we aimed to shed light on potential key intra- and extracellular proteins in reproduction, to form a basis for comparative studies between normal and pathological ovaries and open the door to improved bioengineering techniques for creation of better biomimetic scaffolds.

## EXPERIMENTAL PROCEDURES

### 

#### 

##### Experimental Design and Statistical Rationale

This study involved six 2D-LC/MS experiments using human ovarian cortex from three patients undergoing laparoscopic surgery for benign gynecologic disease. Fresh and cryopreserved samples derived from the same biopsy were analyzed. Each biopsy from each of the patients was initially divided into two fragments; one was immediately analyzed (fresh tissue) and the other was frozen and thawed before analysis (frozen), totaling six experiments. Because the ovarian biopsies were obtained from patients with healthy ovaries, only a small portion of their ovarian tissue was taken (≤9 mm^2^) so as not to impact their ovarian activity. For this reason, we could not carry out technical replicates. Moreover, the scarcity of fresh human ovarian biopsies limited the number of samples available for analysis.

To provide a descriptive draft map of ovarian tissue, a threshold was maintained to select the most confidently detected proteins. This threshold included proteins with a Sequest HT score ≥10 in any of the 6 samples for gene ontology and pathway analyses. Each protein score was calculated by the Proteome Discoverer application as follows: *protein score*, = *(sum of all cross-correlation factors of 0.8 or above)*, + *(peptide charge*, × *peptide relevance factor),*, the default value for the peptide relevance factor being 0.4.

To establish a draft map of the ovarian matrisome, only proteins with a score ≥10 in any of the 6 samples and identified by at least two unique peptides in three or more samples were considered.

Fresh and frozen sample compositions were compared based on spectral counts for each detected matrisome protein. A formal quantitative approach was first attempted to carry out comparisons through a paired *t*, test. However, a number of issues emerged with this type of test, making it hard to draw any conclusions: (1) small sample size because of limited availability of patients; (2) inability to justify the normality of data, possibly warranting use of non-parametric tests; and (3) need for multiple testing, requiring adequate adjustment (inflation) of *p*, values. The first preliminary results pointed to only a few significant (*p*, < 0.05) outcomes (proteins) before adjustment, whereas after adjustment (Benjamini-Hochberg), none of the proteins remained significant. It would therefore be naive to conclude that this somehow proves equivalence. In view of the small sample size and the fact that multiplicity corrections typically suppress detection to avoid false positives, we did not feel that this quantitative argument was sound enough to yield any conclusions. A qualitative, more descriptive approach was therefore pursued, considering the importance of comparing protein content and immunohistochemistry results between fresh and frozen tissues.

##### Collection of Ovarian Tissue and Biopsy Processing

Use of human ovarian cortex was approved by the Institutional Review Board of the Université Catholique de Louvain on November 28, 2016 (IRB reference 2012/23MAR/125, registration number B403201213872). Ovarian biopsies were taken from 3 women (30, 49, and 59 years of age) after obtaining their informed consent. All patients were undergoing laparoscopic surgery for benign gynecologic disease not related to the ovaries (supplemental Table S2). Biopsies were immediately transported on ice to the laboratory in minimal essential medium (MEM)-Glutamax (Gibco, Invitrogen, Merelbeeke, Belgium) and rigorously washed in Dulbecco's phosphate-buffered saline (DPBS) (Gibco) to remove blood remains.

The cortical area of the ovary was selected for analysis, as it is the reservoir of most of the follicle population and would therefore provide the greatest insights into the follicular microenvironment and regulatory factors. Hence, the medullary part of the biopsy was removed, and the cortex was cut into small fragments. A small piece of each biopsy was fixed in formalin and the remaining fragments were either immediately digested or cryopreserved.

##### Tissue Preparation for Fresh Ovarian Cortex

For extraction of intra- and extracellular proteins, including ECM proteins, we applied a modified version of our isolation protocol for human preantral follicles (11). Ovarian cortical fragments were first subjected to mechanical digestion using a tissue chopper (McIlwain Tissue Chopper; Mickle Laboratory, Guilford, UK), before being, incubated with 0.28 Wünsch units/ml Liberase DH (Roche Diagnostics, GmbH, Mannheim, Germany) diluted in 5 ml DPBS in a water bath at 37 °C with mild agitation. After 30 min, the suspension was centrifuged at 260 × *g*, for 15 min at 4 °C, the supernatant was collected, and the action of Liberase was inhibited with 20 mm EDTA (Sigma, Bornem, Belgium). The sample was then stored at −80 °C until MS analysis.

##### Tissue Preparation for Frozen Ovarian Cortex

Freezing of ovarian cortex was performed using our routine protocol (12). Briefly, the ovarian fragments were suspended in a cryoprotective solution consisting of MEM-Glutamax (Gibco) supplemented with 4 mg/ml human serum albumin (HSA, Bornem, Belgium) at 4 °C, and transferred to 2 ml cryovials (Simport, Quebec City, Canada) containing 0.8 ml cryoprotective solution. The cryovials were cooled in a programmable freezer (Freezer Control CL-8800i, CryoLogic, Victoria, Australia) using the following program: (1) cooled from 0 °C to −8 °C at −2 °C/min; (2) seeded manually; (3) cooled to −40 °C at −0.3 °C; and (4) cooled to −140 °C at −30 °C/min before being transferred to liquid nitrogen (−196 °C) for storage. After 24 h in liquid nitrogen, the cryovials were exposed to room temperature for 2 min and immersed in a water bath at 37 °C until the ice completely melted. To remove the cryoprotective solution, the ovarian cortical pieces were immediately transferred from the cryovials to plastic Petri dishes containing MEM, where they were washed three times (5 min per bath). A fragment from each sample was then fixed in formalin and the rest was digested and stored at −80 °C, as described above for fresh tissue.

##### Sample Preparation for Mass Spectrometry Analysis

Total protein content was quantified by the Bradford assay. Three hundred μg of proteins was reduced using 5 mm DTT and incubated at 56 °C for 20 min. After cooling to room temperature, cysteines were alkylated by addition of 30 mm chloroacetamide for 25 min.

Proteins were then precipitated by adding TCA to a final concentration of 10% [w/v]. After centrifugation at 14000 rpm for 5 min at 4 °C, the pellet was resuspended in 100 mm ammonium bicarbonate (pH 8.0) and 0.5 m urea, with continuous vortexing and sonication. Protein digestion was performed using trypsin at an enzyme/substrate ratio of 1:100 [wt/wt] overnight at 30 °C (Promega, Madison, WI). The reaction was halted by adding TFA to a final concentration of 0.1% [v/v] and the sample was stored at −20 °C.

##### Label-free Differential 2D-LC/MS

Tryptic digests were first desalted and concentrated on HyperSep C18 cartridges (50 mg/ml, Thermo Scientific, San Jose, CA) according to the manufacturer's instructions. Peptides were then loaded onto a hydrophilic interaction liquid chromatography (HILIC) TSKgel amide-80 column (4.6 mm by 25 cm; Tosoh Bioscience, Stuttgart, Germany) equilibrated with solvent B (98% ACN [v/v], 0.1% TFA [v/v] in water) and connected to an Agilent 1100 HPLC system. The peptides were separated using a 70-min elution gradient that consisted of 5% to 45% solvent A (2% ACN [v/v], 0.1% TFA [v/v] in water) at a flow rate of 500 μl/min. Absorbance was monitored at 214 nm to ensure that all samples contained similar amounts of material. Fractions were collected at 2-min intervals (starting at 30-min elution, 20 in total) and dried using a Speedvac. Peptides were resuspended in 10 μl of solvent C (3.5% ACN [v/v], 0.1% TFA [v/v] in water) and analyzed by LC-MS/MS. The LC-MS/MS system consisted of an LTQ XL IT mass spectrometer (Thermo Scientific) equipped with a microflow ESI source. Samples (6.5 μl) were injected and desalted on a peptide trap (C18 Pepmap 0.30 × 5 mm, ThermoScientific) equilibrated with solvent C (3.5% ACN [v/v], 0.1% TFA [v/v] in water) at a flow rate of 30 μl/min. After valve switching, peptides were eluted in backflush mode from the trap onto the analytical column (BioBasic C18 0.18 × 150 mm, ThermoScientific) equilibrated in solvent D (5% ACN [v/v], 0.05% formic acid [v/v] in water) and separated using a 70-min gradient from 0% to 70% solvent E (80% ACN [v/v], 0.05% formic acid [v/v] in water) at a flow rate of 1.5 μl/min. The MS scan routine was set to analyze, by MS/MS, the five most intense ions of each full MS scan, with dynamic exclusion enabled to ensure detection of co-eluting peptides.

##### Protein Identification and Quantitation

Protein identification was performed with Sequest HT. More specifically, peak lists were generated by Extract-MSn (Thermo Scientific) within Proteome Discoverer 1.4.2. From raw files, MS/MS spectra were exported using the following settings: peptide mass range 350–5000 Da, minimal total ion intensity 500. The resulting peak lists were searched using Sequest HT against a human protein database obtained from UniProt (March 1, 2014, 87,489 entries). The following parameters were applied: trypsin was selected with proteolytic cleavage only after arginine and lysine; the number of internal cleavage sites was set to 1; mass tolerance for precursors and fragment ions was 1.0 Da; and considered dynamic modifications were +15.99 Da for oxidized methionine and +57.00 for carbamidomethyl cysteine. Peptide matches were filtered using the q-value and posterior error probability calculated by the Percolator algorithm, ensuring an estimated false discovery rate (FDR) below 5%. Filtered Sequest HT output files for each peptide were grouped according to the protein from which they were derived, and their individual peptide spectrum match (PSM) score was taken as an indicator of protein abundance. No normalization procedure was required because sampling rates (*mean PSM*, ÷ *number of detected proteins*,) were similar between all samples (17 ± 0.57 in fresh samples and 16 ± 0.44 in frozen samples). These MS proteomic data were deposited in the ProteomeXchange Consortium database via the PRIDE partner repository with the data set identifier PXD008183.

##### Bioinformatics

Gene ontology (biological process and cellular component) analysis of the ensemble of detected proteins was performed through WebGestalt (http://www.WebGestalt.org) and DAVID (https://david.ncifcrf.gov) online databases. Pathway analysis was conducted via the KEGG pathway (http://www.genome.jp/kegg/pathway.html). Identification of matrisome proteins was achieved by comparison against matrisome atlas proteins (13). JMP^®^ 12.2.0 was used to generate qualitative analysis graphs to compare fresh and frozen samples.

##### Histological and Immunohistochemical Analyses

Histological analysis was performed on fresh and frozen-thawed samples of ovarian cortex. After fixation, the ovarian fragments were dehydrated, embedded in paraffin and serially sectioned (5 μm-thick sections). Every fourth slide was stained with hematoxylin-eosin (Merck, Darmstadt, Germany) for histological evaluation; the other slides (Superfrost® Plus slides, Menzel-Glaser, Braunschweig, Germany) were kept for immunostaining.

Paraffin sections were deparaffinized with Histosafe (Yvsolab SA, Beerse, Belgium) and rehydrated in alcohol series. After blocking endogenous peroxidase activity with 3% H_2_O_2_ [v/v], epitope unmasking was performed with use of citrate buffer (0.01 m) at 98 °C for 75 min in a water bath or Tris-EDTA-Tween buffer (Tris 10 mm, EDTA 1 mm pH 9.0, Tween 20) at 96 °C for 20 min. Slides were incubated for 30 min with 10% goat serum and 1% BSA to block nonspecific binding sites and then analyzed using primary antibodies to proliferating cell nuclear antigen (PCNA) (1/4000 dilution, Dako, Glostrup, Denmark), desmin (1/50 dilution, Dako), alpha-smooth muscle actin (αSMA) (1/100 dilution, Dako), β-catenin (1/15000 dilution, BD Biosciences, San Diego, USA), emilin-1 (1/50 dilution, Sigma), fibrillin-1 (1/100 dilution, Sigma), collagen IV (1/25 dilution, Dako) and VI (1/100 dilution, Acris, Tokyo, Japan).

Immunohistochemical staining was carried out using 3,3′-diaminobenzidine (DAB) horseradish peroxidase chromogen-based system (EnVision^TM^+, Dako) and hematoxylin as a counterstain. Slides were then mounted with DPX mounting medium (Sigma). Negative control samples were obtained by omission of the primary antibody, whereas placenta was used as a positive control for collagen VI, endometrium as a positive control for PCNA, desmin, αSMA, β-catenin and fibrillin-1, and uterine myoma tissue as a positive control for emilin-1 and collagen IV. Glycosaminoglycans were identified by alcian blue staining at pH 2.5 (Bio-Optica, Milan, Italy) to label carboxylated and sulfated proteoglycans (14). Images were taken with a Nikon Eclipse Ci microscope equipped with a Leica DFC450 camera interfaced to Leica Application Suite V4.5 software.

##### Western Blotting

Proteins (25 μg) in each sample were precipitated using the methanol:chloroform method, then boiled in 2X Laemmli buffer and loaded onto polyacrylamide gels. Following SDS-PAGE, the proteins were transferred onto PVDF membranes, which were then blocked in Tris-buffered saline (TBS) containing 0.1% [v/v] Tween and 3% [w/v] BSA. Membranes were incubated overnight at 4 °C with primary antibodies against mimecan (OGN, 1/4000 dilution, Invitrogen, CA, USA) and insulin-like growth factor-binding protein complex acid labile subunit (IGFALS, 1/1000 dilution, OriGene, Rockville, USA), diluted in blocking buffer, then washed extensively in TBS containing 0.1% [v/v] Tween before and after incubation for 1 h with HRP-conjugated secondary antibodies (1/20,000 dilution). Glyceraldehyde-3-phosphate dehydrogenase (GAPDH, 1/10,000 dilution, Merck) was used as a loading control. Immunodetection was achieved with ECL Classico substrate (Merck).

## RESULTS

### 

#### 

##### Total Ovarian Proteome Analysis

A total of 5,253 proteins were detected with an FDR <5%, out of which 1508 unique proteins were confidently identified based on a score ≥10. These 1508 proteins were kept for further bioinformatic analysis.

##### Functional Classification of the Ovarian Proteome and Pathway Analysis

**Fig. 1.**
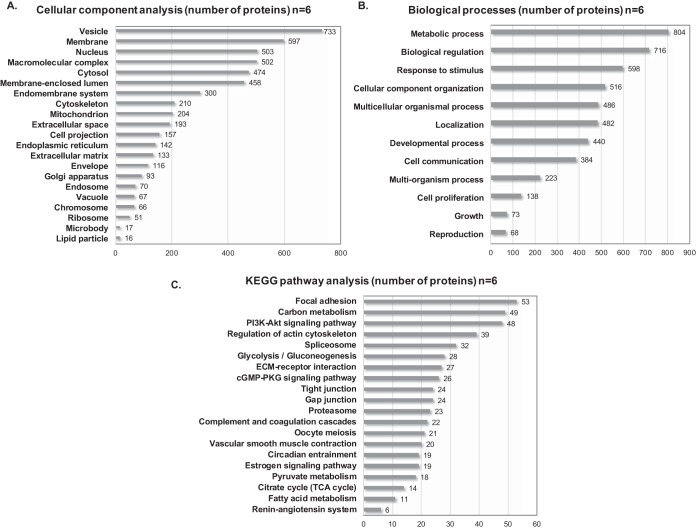
**Gene ontology and pathway analysis of the ovarian proteome.** *A*,, Cellular component analysis. *B*,, Biological process analysis. Enriched biological processes and protein localization were achieved using WebGestalt database. *C*,, KEGG pathway analysis. Proteins were identified based on conversion of their UniProt accession ID to Entrez Gene.

To obtain an extended evaluation of the pool of proteins identified within our samples, we conducted cellular component, biological process and KEGG pathway analyses online using WebGestalt (http://WebGestalt.org) on all detected proteins within the threshold, after converting their UniProt accessions into Entrez Gene and removing duplicates. The following settings were selected to carry out the functional classification: genome protein-coding as a reference set for enrichment analysis, and overrepresentation enrichment analysis (ORA) as a method of reference. We thus obtained a better understanding of the gene ontology of detected proteins within our samples (Fig. 1).

Cellular component analysis revealed, at a glance, the localization of proteins detected by MS and their distribution among cellular compartments (Fig. 1*A*,). It is worth mentioning that whereas membrane proteins represented most of detected proteins, it was also possible to extract proteins from different cellular compartments. Indeed, our protein extraction method enabled us to extract and detect, with a high degree of confidence, extracellular as well as intracellular proteins, transmembrane proteins and cell receptors, such as membrane-associated progesterone receptor component 1, ryanodine receptor 1 and prolow-density lipoprotein receptor-related protein 1.

Biological process analysis showed many detected proteins to be implicated in metabolic processes, growth, proliferation and communication (Fig. 1*B*,). Despite limited available information on ovarian proteomics, 68 proteins were confidently assessed as being involved in reproduction, whereas others were responsive to endogenous and exogenous stimulation, including photosensitive and mechanosensitive proteins.

Relevant biological processes related to reproduction were further investigated through pathway analysis using the KEGG database (Fig. 1*C*,), which highlights proteins involved in the phosphoinositide 3-kinase serine/threonine kinase Akt (PI3K-Akt) signaling pathway and oocyte meiosis pathway. Among our proteomic data, we also detected other pathways linked to ovarian activity, such as cyclic GMP-protein kinase G (cGMP/PKG), gap junctions, and estrogen signaling (supplemental Data S1*A*,).

Different mechano-regulating pathways chiefly related to smooth muscle contraction and relaxation, like the actin cytoskeleton organization pathway, were also identified. Moreover, because cells function in mutual interaction with the surrounding ECM for ECM regulation, cellular adhesion and activity modulation, we detected ECM-interaction receptors and focal adhesion proteins. Interaction between the ECM and ovarian cells is also governed by matrix metalloproteinases (MMPs), which were encountered, but with a very low PSM and score, reflecting their small proportion compared with other proteins found in our samples. Nevertheless, some ECM degradation proteases were clearly discernible, namely cathepsin G, cathepsin D and plasmin.

##### Top 50 Detected Proteins

**Fig. 2.**
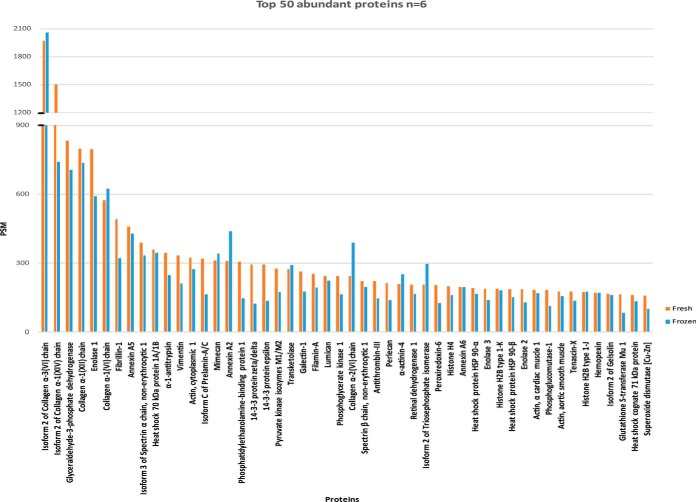
**TOP 50 most abundant proteins.** After retrieval of the top contaminating plasma proteins from the proteome data set, the most abundant proteins in all samples were classified based on their PSM.

High expression of some proteins in the ovary suggested that they may play specific roles in the maintenance of normal ovarian function (Fig. 2). The 50 most abundant proteins observed in our fresh (*n*, = 3) and frozen (*n*, = 3) samples were pooled and ranked based on the number of PSMs after retrieval of hemoglobin subunits and top plasma-contaminating proteins: serum albumin, serotransferrin, ceruloplasmin, transthyretin, immunoglobulin, complement factors and apolipoprotein A-I (inspired by commercial kits available for plasma protein retrieval from analyzable samples). Around 76% of ranked proteins were recorded among the 50 most commonly found proteins in each individual sample. Collagens, as expected, were the most abundant proteins, mainly collagen VI, followed by cytoskeleton organization proteins (vimentin, α-filamin, actin, actinin) and ECM (annexins, fibrillin-1, galectin, lumican) and cellular regulators (GAPDH, transketolase, 14-3-3 protein zeta/delta) from different cellular compartments. Certain proteogylcans were also widely detected, such as mimecan and perlecan.

##### Ovarian ECM Proteome Map

Although gene ontology can offer us a preliminary understanding of the cellular compartments, functions and biological processes of proteins, it has clear limitations with respect to ECM protein differentiation and classification, which is why we needed to clearly establish which proteins should be considered part of the ECM. To this end, all proteins detected by MS were compared against the Matrisome Project data (13) to identify all ECM proteins within our samples. Hence, we were able to provide an extended definition of the ovarian ECM, including not only all structural ECM components, but also proteins able to regulate and remodel the ECM. We subsequently categorized our proteins following Naba *et al.*, 's (2016) classification (13):

1. Core matrisome proteins: ECM glycoproteins, collagens and proteoglycans.
2. ECM-associated proteins.
  - ECM-affiliated proteins: proteins showing biochemical and architectural analogy with ECM proteins or known to be associated.
  - ECM regulators: proteins responsible for ECM turnover.
  - Secreted factors interacting with core ECM proteins.

**Fig. 3.**
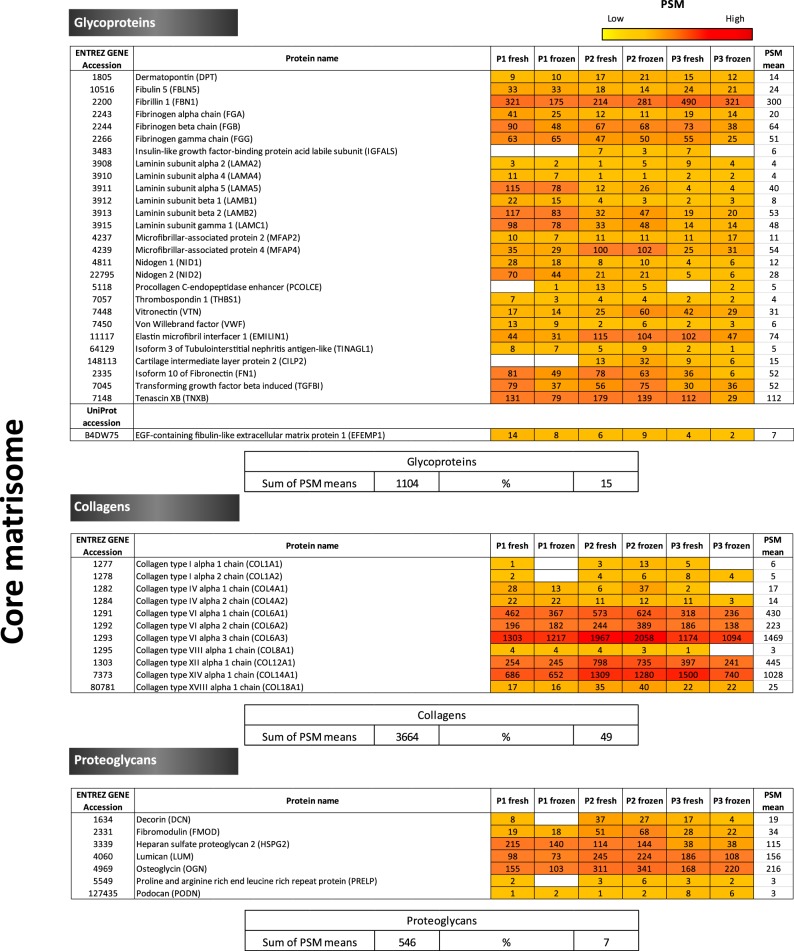

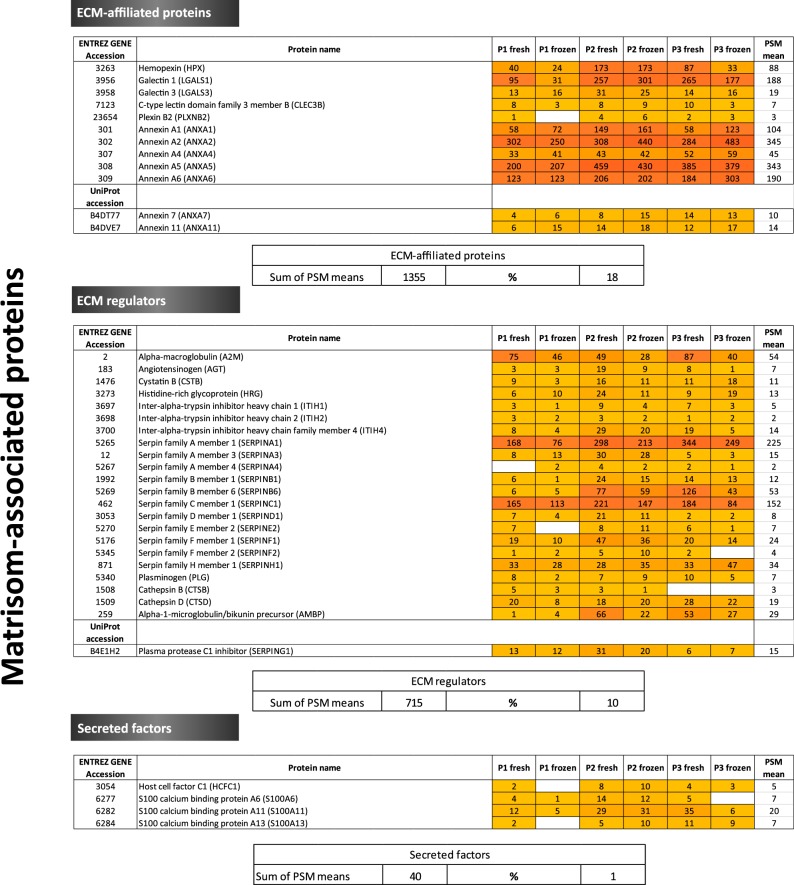
**Ovarian matrisome proteins.** Core matrisome proteins (glycoproteins, collagens and proteoglycans) and matrisome-associated proteins (ECM-affiliated proteins, ECM regulators and secreted factors) detected with a score ≥10 in at least one sample and identified with at least 2 unique peptides in 3 or more samples were taken into consideration. The color code represents protein abundance among fresh and frozen ovarian tissues according to their PSMs. The figure explains how percentages in Fig. 4 were estimated to illustrate matrisome protein distribution in human ovarian tissue (*e.g.*, glycoproteins (%) = (sum of PSM means in detected glycoproteins/total PSM mean sum of all categories)*100 = (1104/7423)*100 ≈ 15%).

**Fig. 4.**
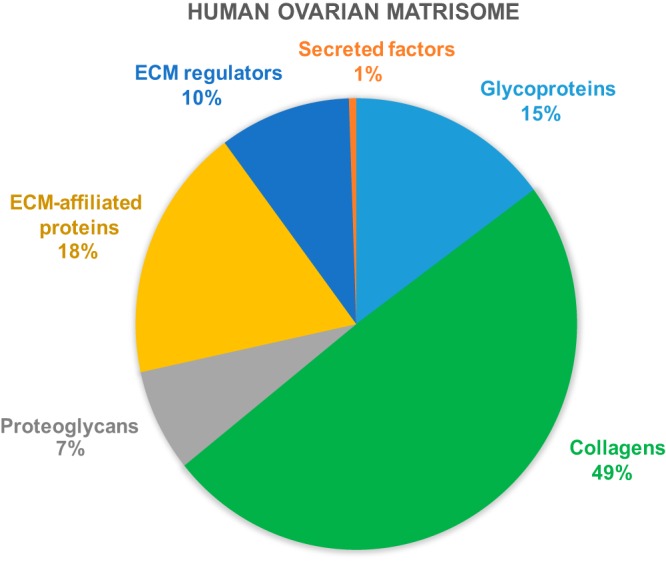
**Pie chart of matrisome protein categories.**

The ensemble above forms the matrisome (Fig. 3). Subsequently, only matrisome proteins that were confidently detected based on score and number of unique peptides were retained and their distribution among samples was evaluated and compared between tests, using their PSM values as an estimate of protein abundance. This approach allowed us to constitute the ovarian matrisome draft map consisting of: (1) the core matrisome, including 28 glycoproteins, 11 subtypes of collagen and 7 proteoglycans, representing 15%, 49% and 7% of the total matrisome proteins respectively (Fig. 3 and 4); and (2) matrisome-associated proteins, including 12 ECM-affiliated proteins, 23 ECM regulators and 4 secreted factors, representing around 18%, 10%, and 1% of the total matrisome proteins respectively. This percentage calculation was based on the sum of PSM means of all proteins within the same category (*e.g.*, collagens), divided by the sum of PSM means of all matrisome proteins (Fig. 3). Thus, the ECM of human ovarian cortex comprises 85 matrisome proteins in total: 46 core matrisome proteins and 39 matrisome associated-proteins.

As expected, secreted factors and ECM modification enzymes were less abundant than structural proteins and, consequently, less well represented in our data set (Fig. 3).

##### Comparison Between Fresh and Frozen-Thawed Ovarian Cortex Proteomic Data

**Fig. 5.**
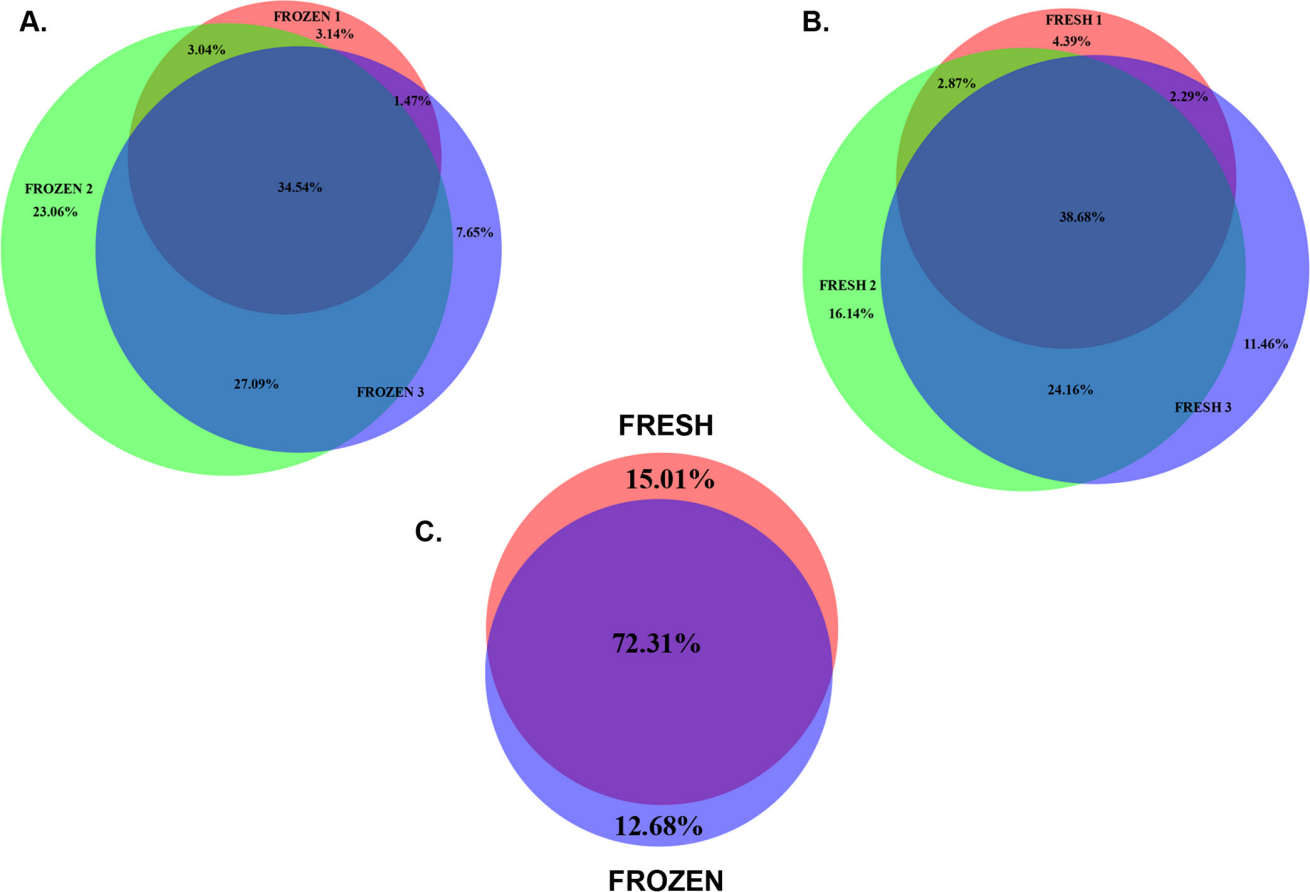
**Fresh and frozen sample comparison showing the overlap of shared and unique proteins among analyzed samples from the three patients.** *A*,, Overlap of fresh samples. *B*,, Overlap of frozen samples. *C*,, Overlap of all fresh and frozen samples. The comparison was made based on the 1508 confidently identified proteins.

**Fig. 6.**
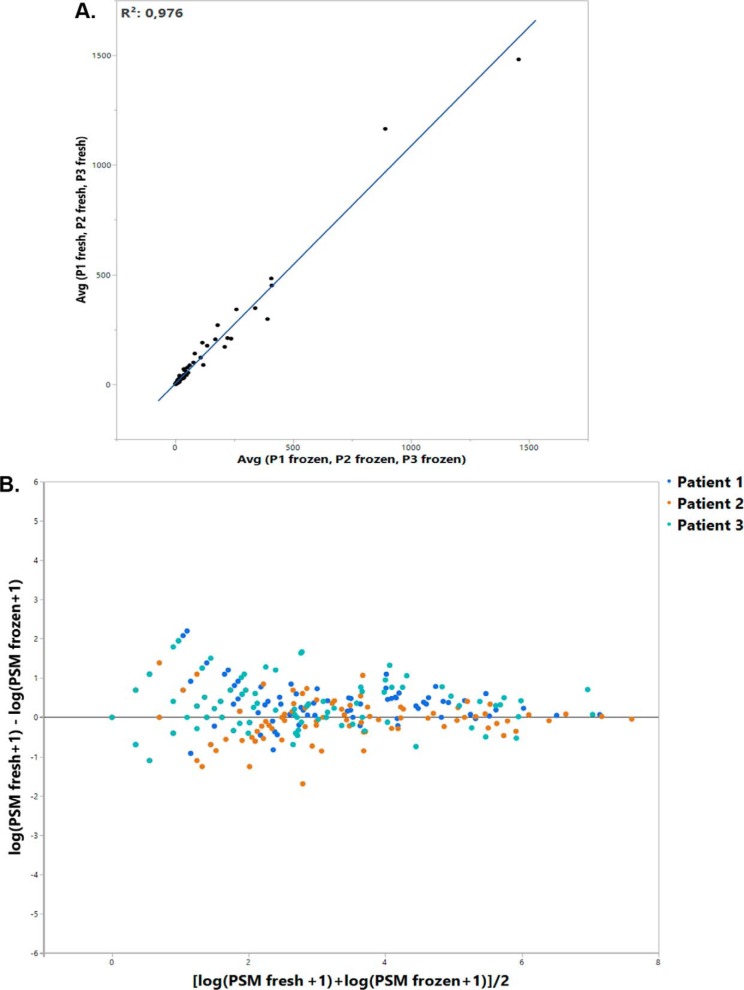
**Comparison of matrisome proteins in fresh and frozen-thawed ovarian cortex samples.** *A*,, Representation of the average abundance (PSMs) of each matrisome protein in both fresh and cryopreserved samples. *B*,, Representation of log(PSM fresh+1) − log(PSM frozen+1) per protein in terms of log(PSM+1) mean in both samples as an abundance indicator. The extent of point deviation from the Y = 0 axis translates the difference in protein presence between fresh and frozen samples.

In order to evaluate the ability of cryopreserved tissue to reflect fresh tissue proteomic composition, we compared proteomic data from fresh and cryopreserved samples. A comparison of total proteins within each group showed similar variability between fresh and frozen samples (Fig. 5*A*,) and, interestingly, an overlap of more than 70% in detected proteins between the two sample types (Fig. 5*B*,). Considering only matrisome proteins, analysis of PSM means revealed no clear difference between fresh and frozen-thawed samples among highly abundant proteins. However, the curve showed some differences among less abundant proteins, which are very difficult to detect by MS in complex samples such as ours (Fig. 6).

##### Histological and Immunohistochemical Analyses

**Fig. 7.**
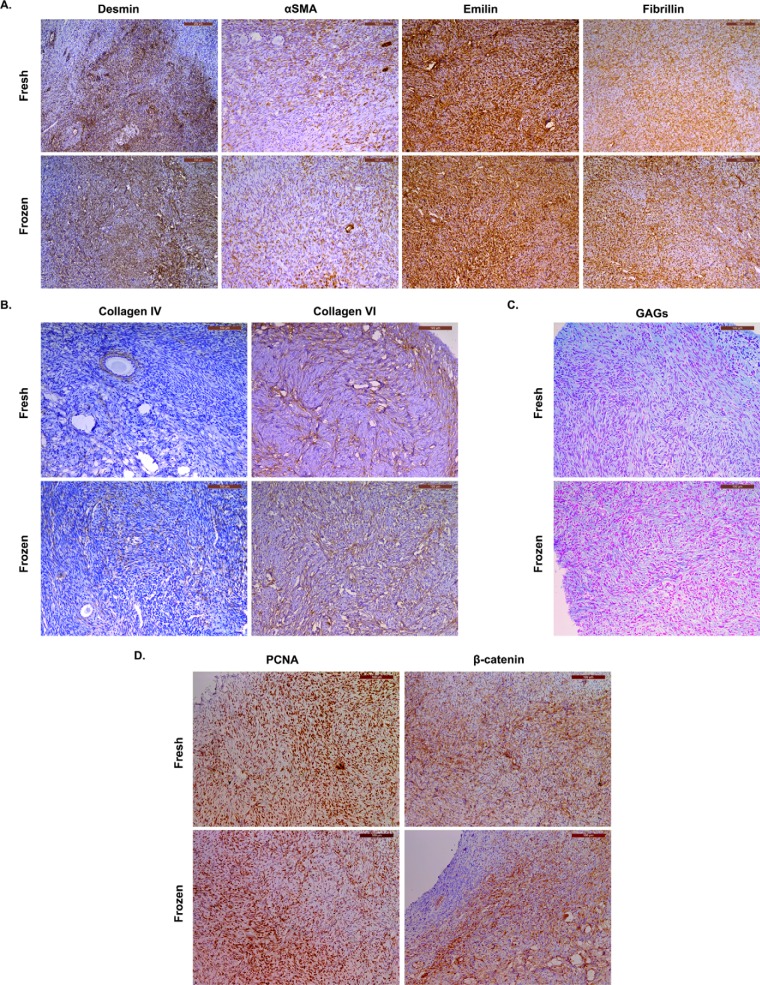
**Immunohistochemical staining in fresh and cryopreserved ovarian cortex.** *A*,, Desmin and αSMA were chosen as muscle contraction markers in the ovary. Emilin-1 and fibrillin-1, both ECM proteins, were selected as elasticity markers. *B*,, Collagen VI, the dominant collagen type in ovarian cortex as demonstrated by MS, was analyzed to confirm proteomic results and compared with collagen IV, the most widely characterized collagen type in ovarian cortex in the literature. *C*,, GAGs were stained by alcian blue at pH = 2.5 to identify carboxylated and sulfated proteoglycans. *D*,, PCNA and β-catenin staining in ovarian cortex are essential proteins for meiosis and cell proliferation.

To assess the localization and distribution of detected proteins of interest by MS, 8 proteins from different cellular components with relevant functions in ovarian tissue were analyzed. Fresh and frozen-thawed samples collected from all patients were evaluated and compared by immunohistochemical staining (Fig. 7). It is important to stress that no visible differences between fresh and frozen-thawed tissue samples were observed in any of the selected proteins.

First, to confirm MS data on the dominant type of collagen in ovarian cortex, collagen VI was stained and compared with collagen IV, because it is the most widely characterized collagen type in the ovary (10-15-17). Positive staining for collagen IV was predominantly found in the follicular basement membrane, whereas collagen VI was detected throughout the interstitial ECM, and often close to the basement membrane. Collagen VI has different roles in tissues where it is expressed, ranging from mechanical roles, which are typical of collagen components of the ECM, to more specific cytoprotective functions, counteracting apoptosis and oxidative damage and regulating cell autophagy and differentiation (18).

Mechanical tissue features related to ovarian cyclic evolvement were evaluated using desmin and αSMA as markers of muscle contraction. Although ovarian cortex was highly immunopositive for desmin, it contained lower levels of αSMA, which was mainly present around blood vessels in all analyzed samples.

ECM proteins emilin-1 and fibrillin-1 were investigated as elasticity markers (19–20). Immunostaining results showed broad distribution of both glycoproteins within ovarian cortex in all patient samples with similar localizations, emphasizing the close relationship between structural and regulatory properties of emilin-1 and fibrillin-1 in connective tissue. Emilin-1 is usually distributed in tissues where resilience and elastic recoil are prominent, is known to interact with integrins, and may connect cells to elastic fibers by providing them with specific cell adhesion features (21); Fibrillin-1 has been shown to provide long-term force-bearing structural support to connective tissues and contains calcium-binding EGF-like domains, integrin-binding Arg-Gly-Asp (RGD) sequences, as well as heparin-binding domains capable of binding cell surface HSPGs (22). The presence of such structural motifs suggests that fibrillin may direct not only cell signaling, but also assembly of elastic microfibers.

Proteins related to meiosis and follicular endowment were also evaluated, namely PCNA and β-catenin as a core molecule of the WNT/β-catenin pathway and major component of cellular junctions involved in cellular communication and signal transduction. Immunohistochemical analysis revealed PCNA-positive cells at the nuclear level in both fresh and frozen samples, as it showed positive β-catenin staining localized in the cell cytoplasm and junctions.

Because glycosaminoglycans are key components of the ECM, ensuring its homeostasis and growth factor sequestering by proteoglycans, their presence was evaluated by alcian blue staining. It revealed their diffuse distribution throughout the ovarian cortex of all patients (Fig. 7).

##### Western Blotting

**Fig. 8.**
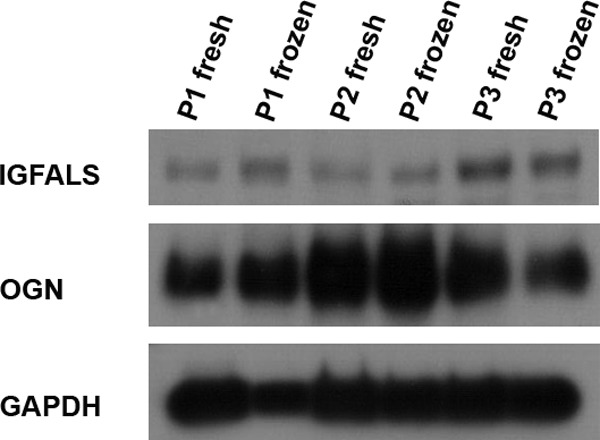
**Western blotting for MS result confirmation.** Some proteins were detected for the first time in ovarian cortex during this study by MS, namely OGN and IGFALS. Western blotting was used to confirm their detection.

Western blotting confirmed detection of two ECM proteoglycans; OGN and IGFALS, in the same samples analyzed by MS (Fig. 8). This is the first time that the presence of these proteins has been reported in human ovarian cortex.

Insulin-like growth factor-binding proteins such as IGFALS are known to play an important role as modulators of insulin-like growth factor (IGF) activity thanks to their high affinity (23). Indeed, they can stimulate or inhibit IGF signaling depending on circumstances by either concentrating IGF close to its receptors or, conversely, hampering sterically their binding (23). This process is of a high importance given the involvement of IGF in follicle growth regulation, selection, atresia, cellular differentiation, steroidogenesis, oocyte maturation, and cumulus expansion evidenced in animal models and humans (24–25).

Western blotting results highlighted the abundance of OGN in ovarian tissue. This protein belongs to a small leucine-rich proteoglycan (SLRP) family, a collagen-associated class of proteins that have an impact on collagen fibrillogenesis and an indirect effect on cell growth. This protein is mainly associated with bone formation and negative regulation of smooth muscle proliferation (26), but its involvement in ovarian tissue is still unknown.

## DISCUSSION

The present study confidently identified 1508 proteins in fresh and frozen human ovarian cortex by 2D-LC/MS. Using bioinformatic tools and data mining analysis, we gained a deeper understanding of the function, cellular distribution and signaling role of these proteins. Although we do not yet fully discern the implication of all of them in the ovarian function, we report the most complete proteomic characterization of human ovarian cortex made to date, including a detailed description of the human ovarian ECM composition, which will lead to a better understanding of the follicle environment.

The technique described can be broadly applied to different tissues of unknown composition to provide a basic understanding of their most abundant proteins and ECM characteristics in one fraction analysis without ECM enrichment. Tissue digestion with Liberase before sample preparation for MS enabled solubilization of major fibrous proteins and thus facilitated analysis of attached molecules and characterization of the ECM. An enzymatic tissue digestion method was chosen because of the high insolubility of ECM proteins, even in strong detergents, which might hinder their detection by MS. Moreover, enzymatic digestion led to rupture of some cellular membranes, which yielded further information on intracellular proteins.

A large panel of proteins was identified within our data set, some of which had only been demonstrated *in vitro*, or in animal models, but never in humans. We therefore turned to gene ontology and pathway analysis to better understand their biological role in ovarian tissue, as well as their interactions.

Fundamental cellular biological processes and several oocyte-related events were detected, namely cell communication pathways involving ECM-receptor interaction, focal adhesion and gap junctions, emphasizing the primordial functional importance of ovarian cell interaction and communication. Focal adhesion proteins made up the second largest group. Proteins included in this category were considered important for ovarian function, because focal adhesion is a key means by which cells sense and respond to the extracellular environment. Conformation of these proteins can vary in response to physical forces, and hence their function (27). This fact can be correlated with the importance of biomechanical regulation mechanisms within the ovary and the impact of the mechanical properties of scaffolds used in 3D culture on ovarian cell fate *in vitro*, (28–29). Thus, greater awareness of ovarian focal adhesion proteins might provide more insights into ovarian cell communication and implication in reproduction. Gap junction channels, another detected protein category, allow direct cell-cell communication and diffusion of fundamental nutrients and chemical cues essential for follicular development (30). Their assembly is promoted by the WNT/β-catenin pathway of gap junction proteins, wherein β-catenin, a protein documented in our MS data and immunohistochemical results, plays a key role in reproduction by influencing estradiol synthesis and adversely affecting follicular development (31–32) (Fig. 7*D*,).

Other pathways of interest were also detected, such as cyclic GMP-protein kinase G (cGMP/PKG) (supplemental Data S1*B*,) implicated in oocyte meiotic arrest (33), and the renin-angiotensin system (RAS) that is presumed to regulate oocyte maturation and quality. Up to day, RAS involvement in hormonal regulation remains unclear because of significant differences between species (34). Hence, by means of our data set acquired by MS, we hope to contribute to the elucidation of unexplored proteins in the RAS pathway in the human ovary (supplemental Data S1*C*,).

Numerous coagulation- and angiogenesis-related proteins were identified and further elucidated by KEGG pathway analysis, such as coagulation factors (*e.g.*, fibrinogen, prothrombin and plasminogen), as well as those linked to the coagulation and platelet-associated regulatory system (*e.g.*, antithrombin, von Willebrand factor, platelet-activating factor acetylhydrolase IB and serpins), revealing their potential function in reproduction. In 2014, Bódis *et al.*, suggested the role of the platelet-associated regulatory system (PARS) in regulating activity of the hypothalamo-hypophyseal ovarian system and its function in inducing and stimulating follicular and oocyte maturation and steroid hormone secretion in the ovary (35). In the light of recent discoveries, coagulation proteins in the ovary appear to occupy new roles beyond plugging blood leakage that go as far as stem cell awakening in the ovary and ovarian rejuvenation (36–37), clearly requiring further investigation.

Although pro-angiogenic factors have been widely explored in the ovary, mainly vascular endothelial growth factor (VEGF), anti-angiogenic factors are still under-investigated. Among identified growth factors, we report detection of pigment epithelium-derived factor (PEDF), a glycoprotein known to have potent physiologic anti-angiogenic activity that negates VEGF action (38), and thus plays a potential anti-tumoral role (39). PEDF may also function as a gonadal protectant thanks to its anti-inflammatory and anti-oxidative abilities (40–41), which are two important functions in reproduction, considering that oxidative stress and inflammation have been correlated with infertility in women (38). Moreover, high levels of PEDF secreted before ovulation may induce apoptosis in ovarian surface epithelium cells surrounding the follicle to facilitate release of oocyte (42).

Other rarely explored proteins in human ovaries were documented in our data set, namely 14-3-3 protein isoforms delta and epsilon, which are among the top 50 most abundant proteins. 14-3-3 proteins are known to be central mediators modifying cell-signaling processes, including cell cycle regulation and apoptosis (43–46). It has been suggested that these proteins are involved, at least in the *Xenopus*, genus, in maintaining prophase I arrest in germinal vesicle-intact oocytes by sequestering the key phosphatase, in meiosis resumption M-phase inducer phosphatase 2 (CDC25B), in an inactive state (47).

In view of the lack of knowledge of the follicular environment, particularly ECM proteins, this study provides the most comprehensive description available of healthy human ovarian ECM. ECM protein recognition in raw MS data was achieved by comparison of confidently detected proteins with the Matrisome Project data set (13), an *in silico*, identified ECM protein set, founded on the characteristic domain-based organization of ECM proteins (48). Hence, we were able to define not only the ovarian ECM, but also the matrisome: an extended definition of the ECM and associated proteins. The dominant collagen type in the ovaries was revealed to be collagen VI, which we showed to be ubiquitously expressed throughout the ECM by immunohistochemistry. It is a basement membrane-anchoring molecule that interacts with collagen IV and may have additional cytoprotective and regulatory functions (18). In addition, it has been suggested that type VI collagen microfibrils are resistant to MMPs but are susceptible to degradation by serine proteases, which are enzymes secreted by granulocytes and neutrophils and known to be present in the ovary during the inflammatory phase preceding ovulation (49).

Within the category of glycoproteins, we identified laminin, fibrillin and thrombospondin, fundamental proteins of the basement membrane, of key importance for cellular attachment, cell proliferation and ECM organization. Most importantly, however, they share EGF-like intrinsic domains, which might bind to EGF receptors and modulate its signaling following their release by ECM proteolysis.

Within the category of proteoglycans, perlecan (HSPG2) was of interest. Like many proteoglycans, HSPG2 is able to bind growth factors and cytokines and sequester them in the ECM and may be crucially important in the action of basic fibroblast growth factor (bFGF), a key growth factor with pro-angiogenic and anti-apoptotic effects in the ovary. Localized in the basement membrane, HSPG2 provides a barrier, which is both size- and charge-selective, and promotes cell adhesion, endothelial cell growth and regeneration. In the ovary, it has been identified as a major estrogen-binding protein in follicular fluid (50).

Another proteoglycan that was unexpectedly detected is OGN, also known as osteoglycin. Its down-regulation in vascular smooth muscle cells results in an increased cell proliferation (51), which can provide insights into possible vascular stimulation of the ovarian tissue grafting site by controlling OGN expression.

In osteoblasts, bone morphogenetic protein 2 (BMP-2) increases OGN expression, whereas in the ovary, the same protein is implicated in primordial follicle assembly during fetal life (52), which might suggest the possible involvement of OGN in ovarian fetal remodeling and follicular assembly under the action of BMP-2. Further research is nevertheless needed to elucidate its function in the ovary, especially in adult life.

In the ovary, enzymatic ECM degradation is required to allow activated follicle expansion. However, to protect surrounding cells from proteolysis, different protease inhibitors appear to be secreted in the ovary, such as protein AMBP, alpha-2-macroglobulin and serpins. The latter is the most widely detected ECM regulator protein family and its expression is closely related to ovarian function (53). Indeed, folliculogenesis stage-specific expression of serpins seems to participate in the growth and atresia of follicles (54), as it may control ECM remodeling (55).

Most of ECM-affiliated proteins have seldom been studied, especially in the human ovary, where we detected galectin-1 and galectin-3. Galectins are a family of β-galactoside-binding proteins implicated in modulating cell-cell and cell-matrix interactions. In 2004, Walzel H *et al.*, demonstrated the inhibitory effect of galectin-1 on the steroidogenic activity of granulosa cells, interfering with hormone-receptor interaction and resulting in decreased responses to FSH stimulation in pigs (56). Although galectin-3 has similar functions to galectin-1, it has been associated with loss of progesterone synthesis in the mouse ovary, showing increased presence in atretic preantral follicles and the later stages of luteolysis (57). This protein been described in a variety of tissues, but not explicitly in healthy human ovarian cortex. It plays a role in diverse biological events, such as embryogenesis, angiogenesis, adhesion, cellular proliferation, apoptosis and modulation of immunity and inflammatory processes (58). Overexpression of both galectins is related to ovarian carcinoma; therefore, unraveling the regulatory mechanisms might provide therapeutic solutions.

The third category of matrisome-associated proteins includes ECM-secreted factors. Proteins in this group were the least abundant, as expected, but several S100 proteins emerged as the most abundant protein type in this category. In fact, binding of calcium to the S100 protein causes structural rearrangement, exposing a target-binding surface. Target-binding results in a range of responses, from inflammation (S100A13) and cytoskeletal reorganization (S100A10), to cell growth control (S100A9) and tumor suppression (59–60). However, little is known about the action of these calcium-binding proteins in the ovary.

To our knowledge, this is the first time that fresh human ovarian cortex has been analyzed by MS and compared with frozen tissue cryopreserved using the protocol that has so far generated 13 live births after transplantation in our hospital. This comparison demonstrates the suitability of cryopreserved tissue to accurately represent the proteomic composition of fresh tissue, proving that it can be used to conduct more extended MS analyses.

In conclusion, our study provides an accurate first draft map of human ovarian cortex, with identification of its ECM proteins. It represents the first step in human ovary characterization, essential for development of a biomimetic artificial ovary and greater understanding of fertility in women.

## DATA AVAILABILITY

Raw MS proteomic data were deposited in the ProteomeXchange Consortium database via the PRIDE partner repository with the data set identifier PXD008183 (https://www.ebi.ac.uk/pride/archive/projects/PXD008183).

## Supplementary Material

Supplemental data

Detected proteins in all analyzed samples

## Footnotes

^1^ The abbreviations used are:

ECM: extracellular matrix
2D-LC/MS: two-dimensional liquid chromatography mass spectrometry
bFGF: basic fibroblast growth factor
BMP-2: bone morphogenetic protein 2
CGMP-PKG: cyclic GMP-protein kinase G
DAB: 3,3′-diaminobenzidine
EGF: epithelial growth factor
FDR: false discovery rate
GAPDH: glyceraldehyde-3-phosphate dehydrogenase
HILIC: hydrophilic interaction liquid chromatography
HSA: human serum albumin
HSPG2: perlecan
IGF: insulin-like growth factor
IGFALS: insulin-like growth factor-binding protein acid-labile subunit
MEM: minimal essential medium
MMP: matrix metalloproteinase
OGN: mimecan
ORA: overrepresentation enrichment analysis
PARS: platelet-associated regulatory system
PCNA: proliferating cell nuclear antigen
PEDF: pigment epithelium-derived factor
PI3K-Akt: phosphoinositide 3-kinase serine/threonine kinase Akt
PSM: peptide spectrum match
RAS: renin-angiotensin system
SLRP: small leucine-rich proteoglycan
TBS: tris-buffered saline
VEGF: vascular endothelial growth factor
αSMA: alpha-smooth muscle actin.
