# Supplementary material for: A Draft Map of the Human Ovarian Proteome for Tissue Engineering and Clinical Applications
Source: Mol Cell Proteomics. 2018 Feb 23;18(Suppl 1):S159–73. doi: 10.1074/mcp.RA117.000469 (PMC6427241; doi:10.1074/mcp.RA117.000469)
Supplement: Supplemental data [file 133897_1_supp_76620_p4744r.pdf]

Supplemental figures

S1. Pathway diagrams of estrogen signaling, RAS and cGMP/PKG

Proteins detected by MS were mapped using the KEGG database to understand their interaction and elucidate their functions. Identified proteins within **a)** the estrogen signaling pathway, **b)** cyclic GMP-protein kinase G (cGMP/PKG) and **c)** the renin angiotensin system (RAS) are marked with a red star.

a.

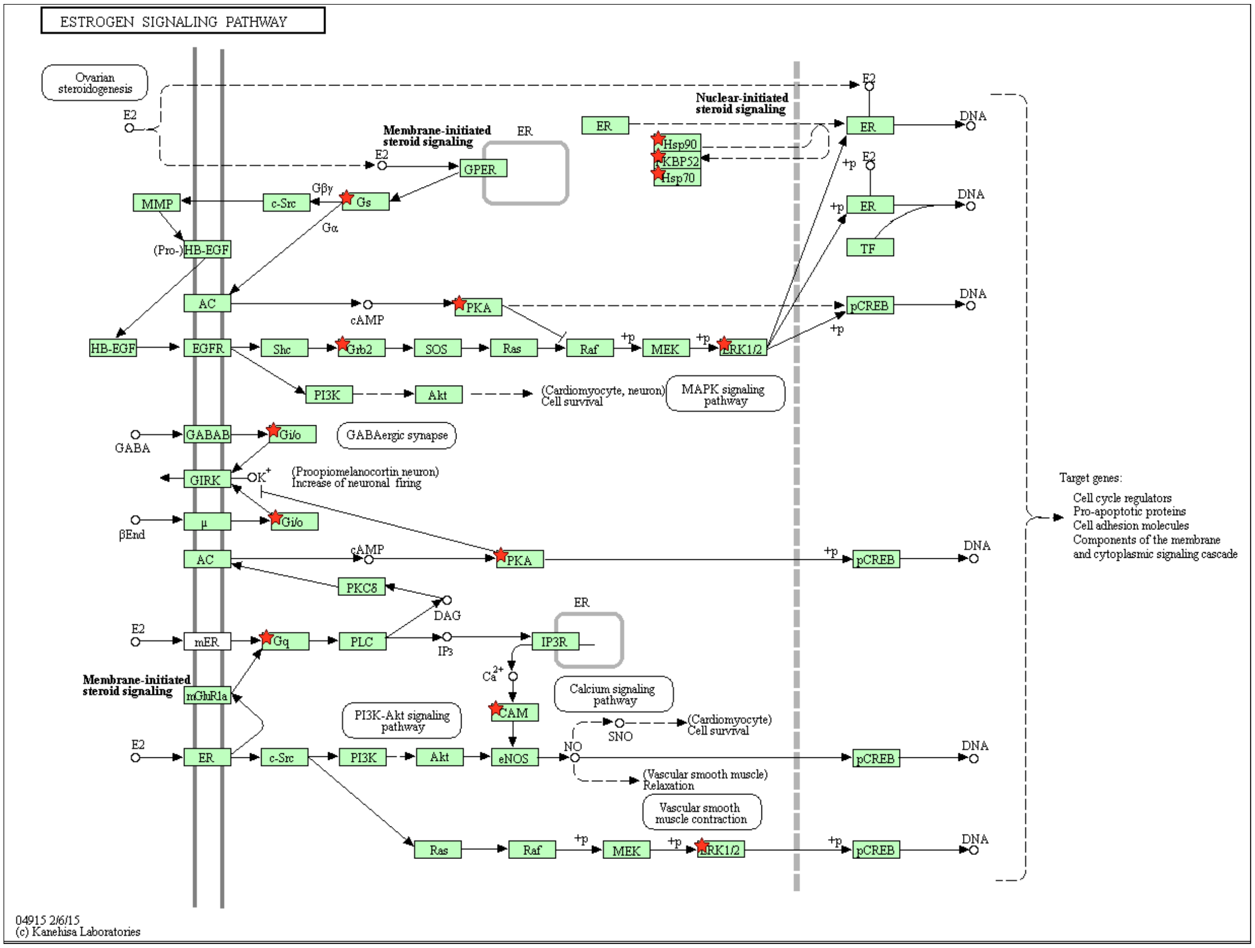

b.

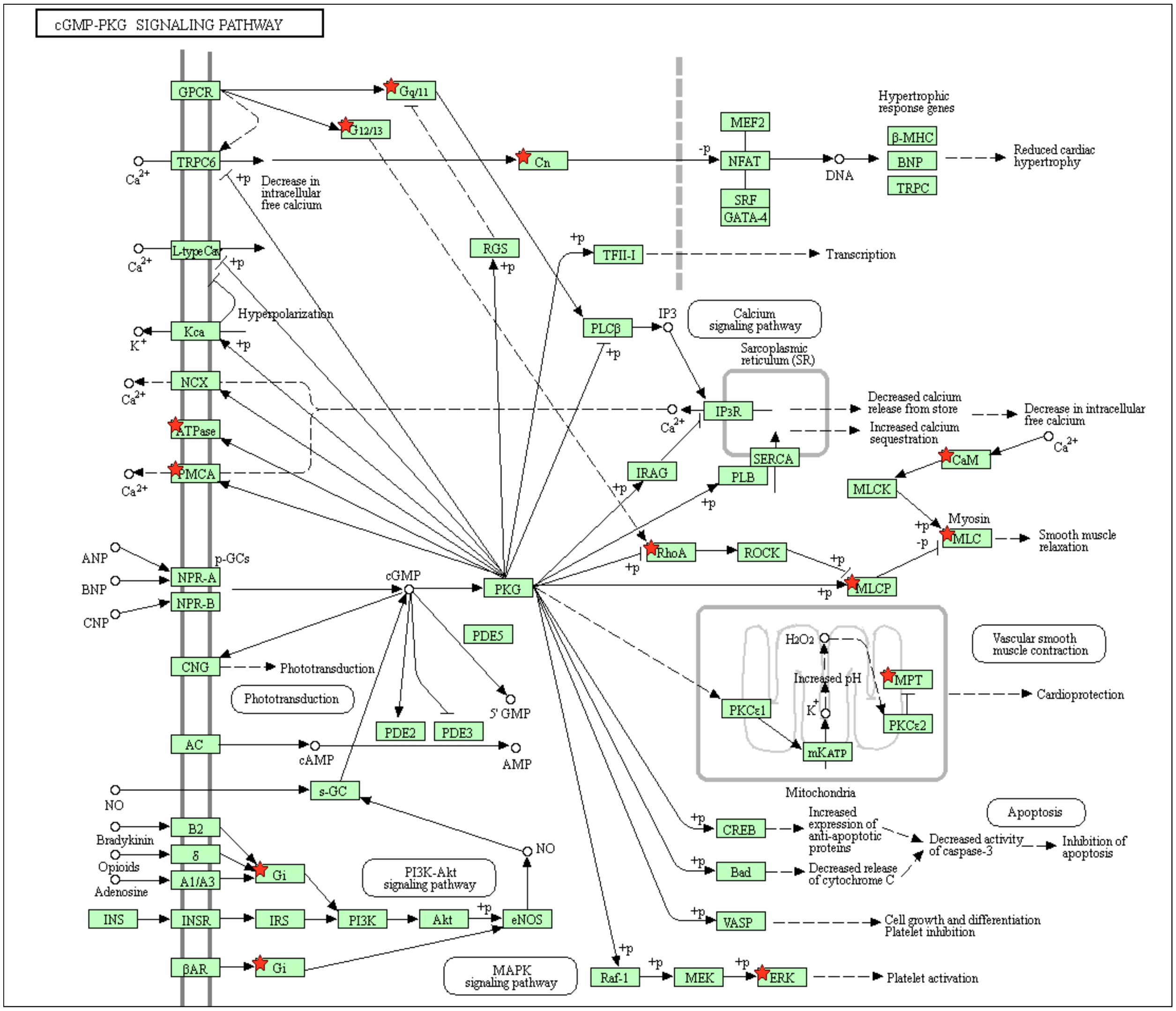
cGMP-PKG SIGNALING PATHWAY
GPCR
Gq/11
G12/13
TRPC6
Cn
MEF2
NFAT
SRF
GATA-4
DNA
Hypertrophic response genes
β-MHC
BNP
TRPC
Reduced cardiac hypertrophy
Decrease in intracellular free calcium
L-type Ca
RGS
TFII-I
Transcription
Kca
Hyperpolarization
PLCβ
IP3
Calcium signaling pathway
Sarcoplasmic reticulum (SR)
NCX
IP3R
Decreased calcium release from store
Decrease in intracellular free calcium
ATPase
Increased calcium sequestration
PMCA
IRAG
SERCA
PLB
CaM
MLCK
Myosin
ANP
p-GCs
NPR-A
BNP
NPR-B
CNP
cGMP
PKG
RhoA
ROCK
MLC
Smooth muscle relaxation
MLCP
PDE5
CNG
Phototransduction
5' GMP
PDE2
PDE3
AC
cAMP
AMP
Vascular smooth muscle contraction
H2O2
Increased pH
MPT
Cardioprotection
PKCε1
PKCε2
mKATP
Mitochondria
NO
s-GC
B2
Bradykinin
δ
Opioids
A1/A3
Adenosine
Gi
PI3K-Akt signaling pathway
INS
INSR
IRS
PI3K
Akt
eNOS
CREB
Increased expression of anti-apoptotic proteins
Apoptosis
Decreased activity of caspase-3
Inhibition of apoptosis
Bad
Decreased release of cytochrome C
VASP
Cell growth and differentiation
Platelet inhibition
βAR
MAPK signaling pathway
Raf-1
MEK
ERK
Platelet activation

c.

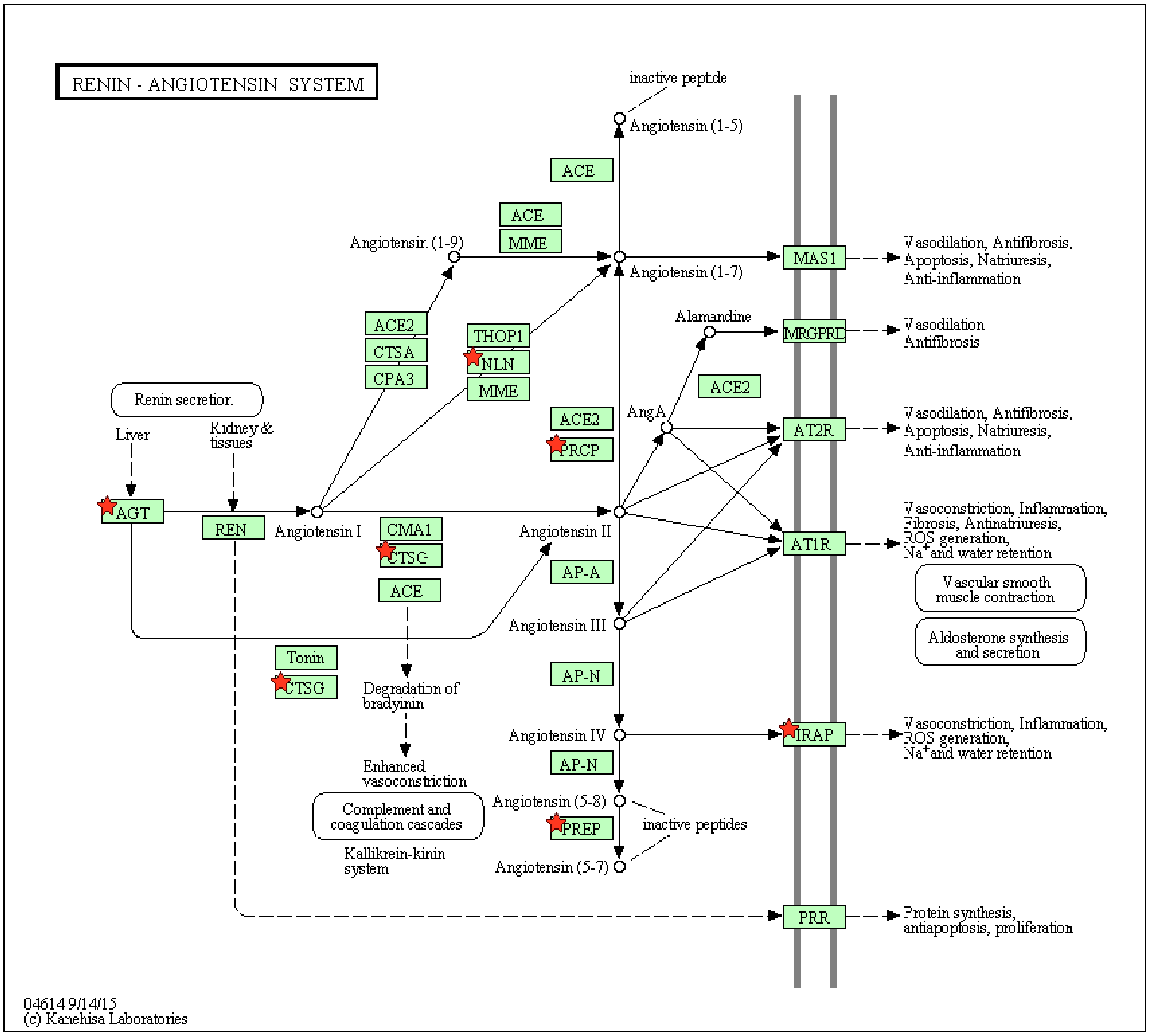
RENIN - ANGIOTENSIN SYSTEM
inactive peptide
Angiotensin (1-5)
ACE
ACE
MME
Angiotensin (1-9)
Angiotensin (1-7)
MAS1
Vasodilation, Antifibrosis, Apoptosis, Natriuresis, Anti-inflammation
ACE2
CTSA
CPA3
THOP1
NLN
MME
Alamandine
MRGPRD
Vasodilation Antifibrosis
ACE2
Renin secretion
Liver
Kidney & tissues
ACE2
PRCP
AngA
AT2R
Vasodilation, Antifibrosis, Apoptosis, Natriuresis, Anti-inflammation
AGT
REN
Angiotensin I
CMA1
CTSG
Angiotensin II
AT1R
Vasoconstriction, Inflammation, Fibrosis, Antinatriuresis, ROS generation, Na+ and water retention
Vascular smooth muscle contraction
Aldosterone synthesis and secretion
ACE
AP-A
Angiotensin III
Tonin
CTSG
Degradation of bradyinin
AP-N
Angiotensin IV
IRAP
Vasoconstriction, Inflammation, ROS generation, Na+ and water retention
Enhanced vasoconstriction
AP-N
Complement and coagulation cascades
Angiotensin (5-8)
PREP
inactive peptides
Kallikrein-kinin system
Angiotensin (5-7)
PRR
Protein synthesis, antiapoptosis, proliferation
04614 9/14/15
(c) Kanehisa Laboratories

S2. Clinical characteristics of ovarian tissue donor patients

	Patients		
	P1	P2	P3
**Age**	30	49	59
**Number of live births**	0	2	2
**Number of abortions**	0	1	2
**Gynecologic disease**	Peritoneal endometriosis	Menometrorrhagia	BRCA1 mutation
**ICD-10[1] code**	N80.3	N92	Z15.01
**Clinical stage**	-	-	pTis (DCIS)[2] pN0[3]
**Surgery**	Unilateral salpingectomy by laparoscopy	Radical hysterectomy by laparoscopy	Radical hysterectomy by laparoscopy
**Histology evaluation**	Paramesonephric paratubal cyst; left hydrosalpinx associated with endometriotic tubular lesions; no sign of malignancy; no ovarian samples for analysis (ovaries not affected by the disease, as observed by the surgeon)	Bulky submucous uterine leiomyoma; superficial uterine adenomyosis; menstrual endometrium; normal ovaries; no sign of malignancy	Atrophic endometrium and uterine adenomyosis; normal cervix and ovaries; no sign of malignancy
**Hormonal treatment**	Zoladex	NO	NO

[1]ICD-10 code: International Statistical Classification of Diseases and Related Health Problems, 10th Revision

[2]pTis (DCIS): Ductal carcinoma in situ

[3]pN0: No regional lymph node metastasis identified
